# Supplementary material for: A Novel Hemocyte-Derived Peptide and Its Possible Roles in Immune Response of Ciona intestinalis Type A
Source: Int J Mol Sci. 2024 Feb 6;25(4):1979. doi: 10.3390/ijms25041979 (PMC10888236; doi:10.3390/ijms25041979)
Supplement: Supplementary file 1 [file ijms-25-01979-s001.zip › ijms-2806734 Figures S1 and S2_proofreading.pdf]

A

>KY21.Chr12.349
MDRKIVFALLLLMSVQVSMAFHW**NERKGAEPQFPPEM**GDEIAADA
ERLMRKAAKDHWSNKMAKDVIWWEQ

B

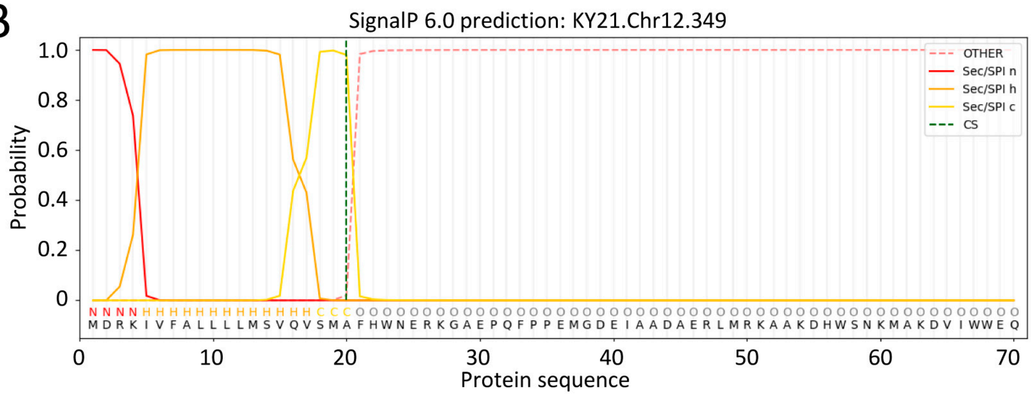

C

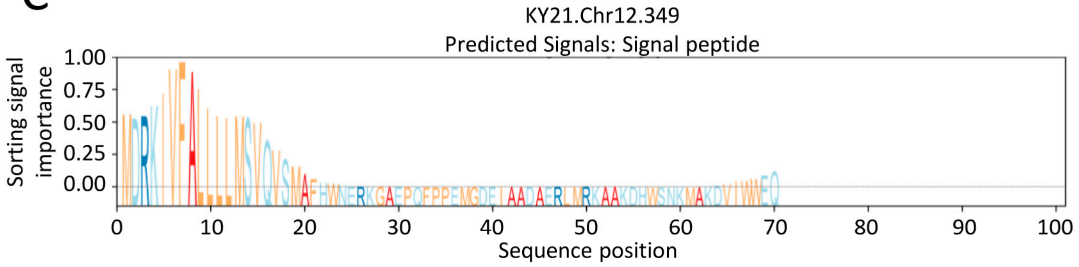

Localizations	Signals	Cytoplasm	Nucleus	Extracellular	Cell membrane
Extracellular	Signal peptide	0.116	0.025	**0.958**	0.038
**Mitochondrion**	**Plastid**	**Endoplasmic reticulum**	**Lysosome/ Vacuole**	**Golgi apparatus**	**Peroxisome**
0.044	0.004	0.072	0.163	0.122	0.002

Figure S1: *In silico* analyses of the precursor sequence of CiEMa. (A) The translated sequence of the *CiEma* gene from the Ghost database is shown. The predicted N-terminal signal sequence is marked in grey, and the mature peptide identified in Figure 1 is shown in red. (B) The N-terminal signal sequence was predicted using SignalP 6.0. The peptide bond between Ala20 and Phe21 is suggested to be the cleavage site. (C) Subcellular localization of CiEMa was predicted using DeepLoc 2.0. The N-terminal sequence was predicted to serve as a signal peptide (upper panel), and CiEMa was predicted to be secreted into the extracellular region (lower panel).

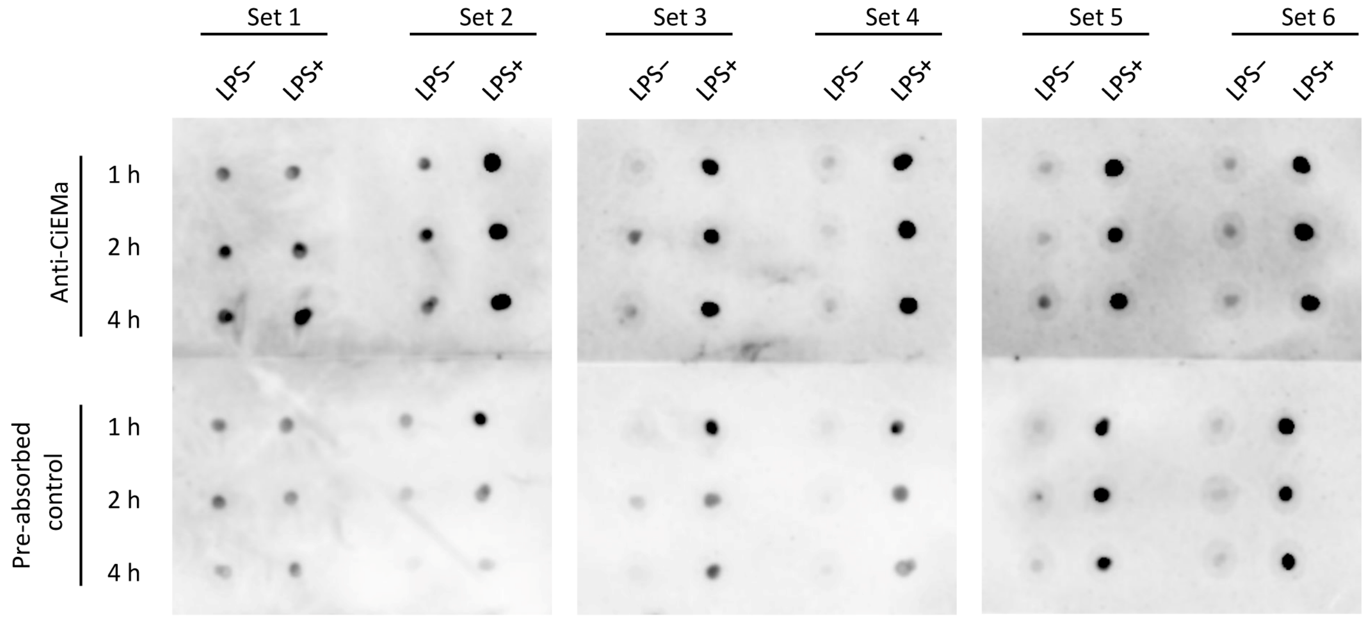

Figure S2: Original images of dot blot analyses in Figure 7B. Six independent data sets using anti-CiEMa antibody and pre-absorbed controls are shown.
