# Supplementary material for: A Novel Hemocyte-Derived Peptide and Its Possible Roles in Immune Response of Ciona intestinalis Type A
Source: Int J Mol Sci. 2024 Feb 6;25(4):1979. doi: 10.3390/ijms25041979 (PMC10888236; doi:10.3390/ijms25041979)
Supplement: Supplementary file 1 [file ijms-25-01979-s001.zip › Table S2_rev.pdf]

Table S2 RNA-seq of the CiEMa-stimulated *Ciona* pharynx

Sample	Total reads	% mapped	Accession
Pha_CiEMa_0h	70,659,570	84.93	SRR26963725
Pha_CiEMa_1h	80,794,444	85.50	SRR26963724
Pha_CiEMa_2h	84,126,938	85.08	SRR26963723
Pha_CiEMa_4h	84,462,016	84.30	SRR26963722
Pha_CiEMa_8h	109,996,394	87.07	SRR26963721
