# Supplementary figures and images for: A Novel Hemocyte-Derived Peptide and Its Possible Roles in Immune Response of Ciona intestinalis Type A
Source: Int J Mol Sci. 2024 Feb 6;25(4):1979. doi: 10.3390/ijms25041979 (PMC10888236; doi:10.3390/ijms25041979)

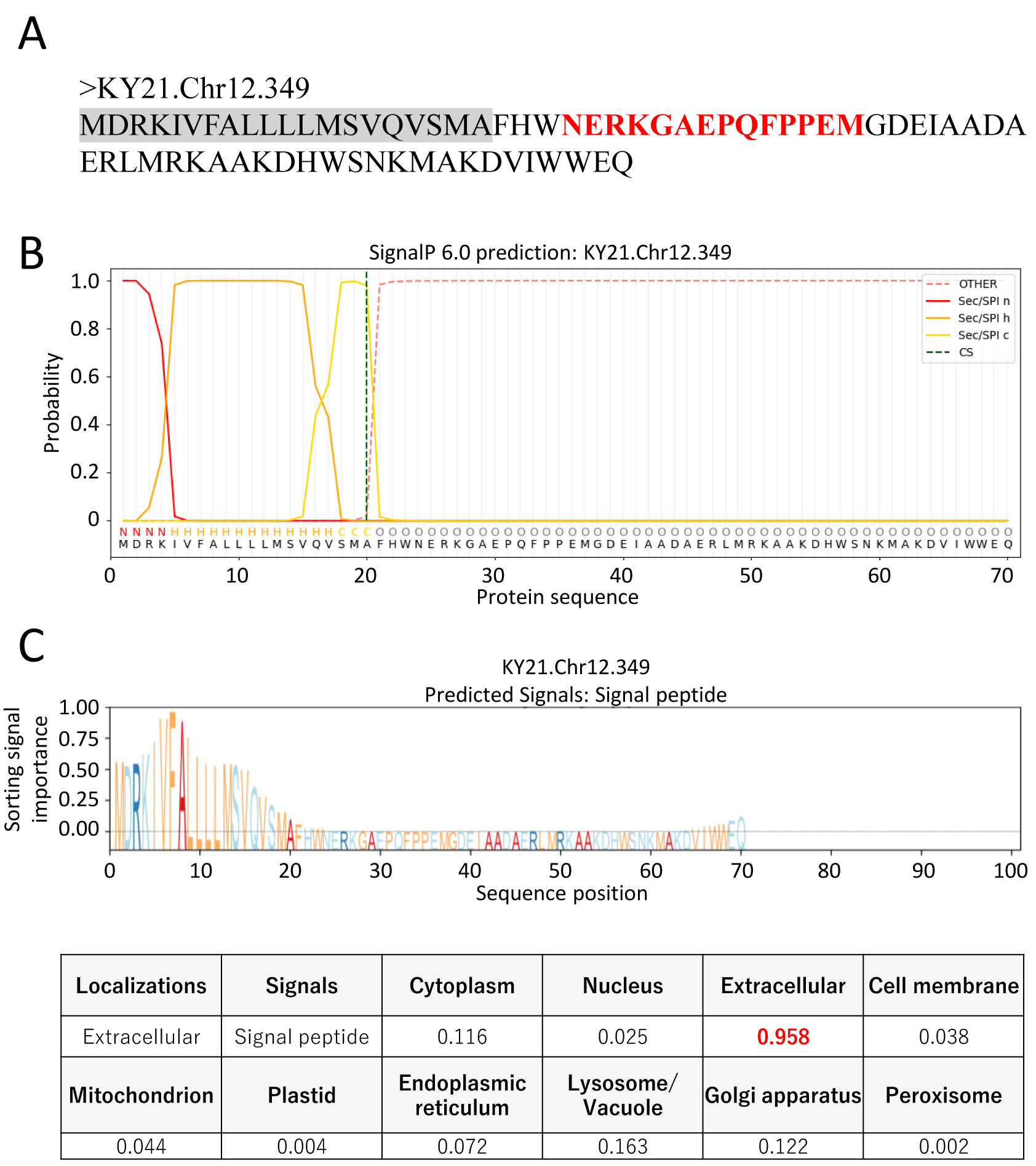

Supplement: Supplementary file 1 [file ijms-25-01979-s001.zip › Figure S1.tif]

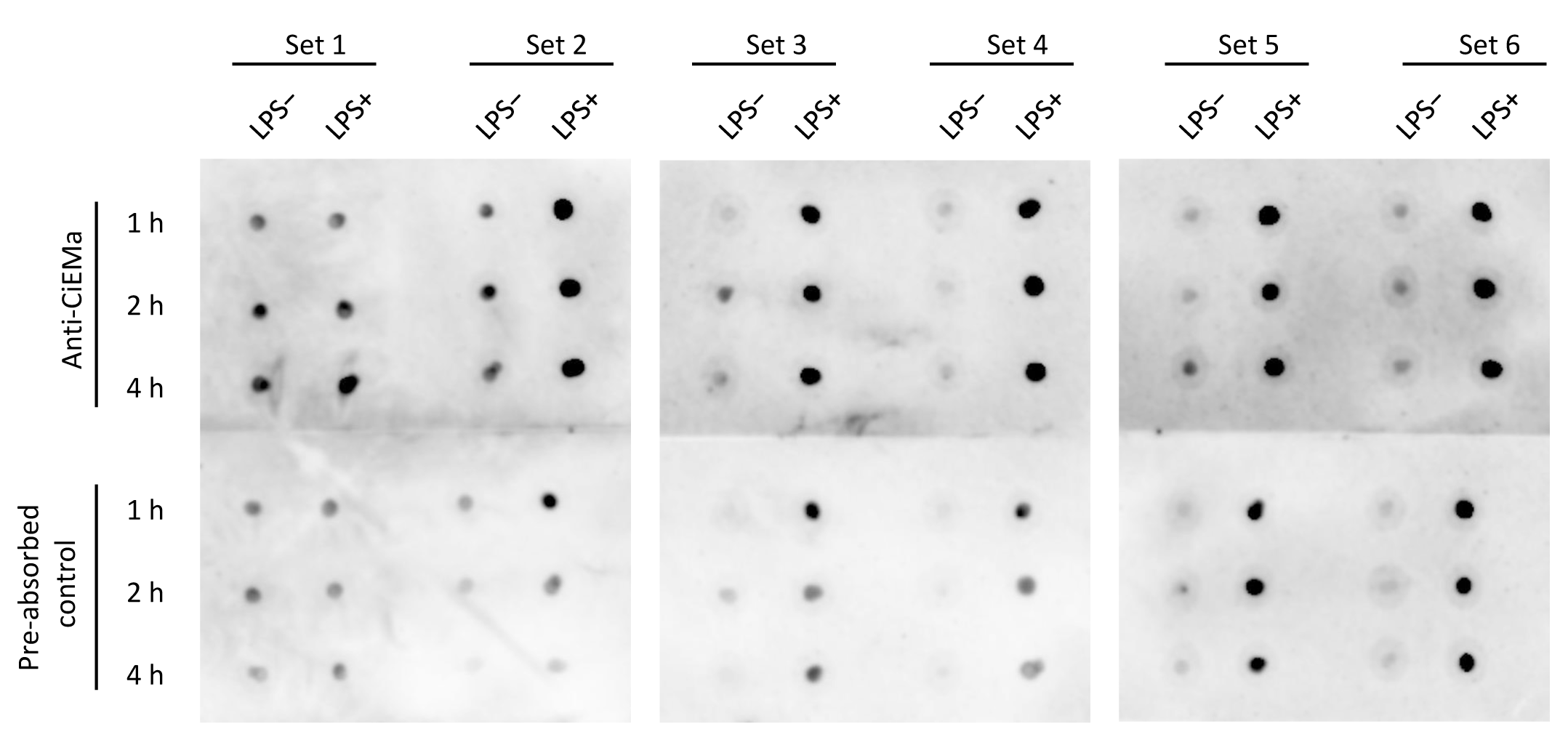

Supplement: Supplementary file 1 [file ijms-25-01979-s001.zip › Figure S2_rev.tif]
